# Personalized Medicine: Recent Progress in Cancer Therapy

**DOI:** 10.3390/cancers13020242

**Published:** 2021-01-11

**Authors:** Ann Hoeben, Elbert A. J. Joosten, Marieke H. J. van den Beuken-van Everdingen

**Affiliations:** 1 Department of Internal Medicine, Division of Medical Oncology, GROW—School for Oncology and Developmental Biology, Maastricht University Medical Center, 6229 HX Maastricht, The Netherlands; 2 Department of Anesthesiology and Pain Management, Maastricht University Medical Center, 6229 HX Maastricht, The Netherlands; bert.joosten@mumc.nl; 3 Centre of Expertise for Palliative Care, Maastricht University Medical Centre, 6229 HX Maastricht, The Netherlands; m.vanden.beuken@mumc.nl

Personalized medicine (PM) or precision medicine in oncology is an emerging approach for tumor treatment and prevention that takes into account inter- and intra-tumor variability in genes, tumor (immune) environment, and lifestyle and morbidities of each person diagnosed with cancer. PM has the potential to tailor therapy towards the oncogenic drivers of the tumor and modulate the tumor immune environment. Furthermore, PM aims to optimize tumor response, thereby taking into account the therapy-induced toxicities for each specific patient. In this way, optimized tumor response is combined with the preservation of organ function and, thus, quality of life. Moreover, this ensures better patient care in the end, which is, of course, what it aims for.

In this context, this present Special Issue of *Cancers* aims to discuss and present the latest research on precision medicine and recent advances in personalized medicine in cancer and its accompanying symptoms.

First of all, Gambardella and colleagues [1] review the development of targeted therapies over the past decades for those tumors displaying a unique oncogenic driver. They emphasize the need for a multi-omics approach, integrating DNA and RNA alterations, allowing a better understanding of tumor biology, intra-tumor heterogeneity, and the development of immune-defense mechanisms. This allows for the identification of tumor-specific biomarkers as well as the development of innovative treatment strategies optimizing response rates and circumventing therapy resistance. Gambarella et al. also touch upon the evolving field of liquid biopsies, a non-invasive way to detect and diagnose cancers and the early development of treatment resistance.

Colorectal cancer is a frequently used and well-known model in which tumor-tailored treatment has already been implemented. In colorectal cancers, the stepwise accumulation of genetic and epigenetic events leading to carcinoma formation is well known. This has identified prognostic and predictive biomarkers such as KRAS (Kirsten Rat Sarcoma virus) and microsatellite instability (MSI), guiding targeted treatment choices in the current standard of care. In this Special Issue, Koulis et al. [2] summarize emerging biomarkers and innovative liquid biopsy platforms that may pave the way towards novel combination treatment options, in which not only tumor cells are targeted but also the tumor microenvironment. Moreover, in non-small cell lung cancers (NSCLCs), driver mutations are being identified for which successful treatment strategies have been developed. Ferrara et al. [3] describe known oncogenic drivers in NSCLC and the different targeted treatment strategies that have been shown to improve the overall survival of these patients. Since only a minority of NSCLC express programs currently know oncogenic drivers, the authors also conclude that further research is needed to identify new molecular targets. To optimize patient- and tumor-tailored treatments in patients with breast cancer, Mazo et al. [4] present an in silico analysis in which they show that four well-known numeric risk scores (OncoMasTR, EndoPredict, OncotypeDX, and tumor-infiltrating leucocytes) were significantly predictive of pathological complete remission to neo-adjuvant chemotherapy in Estrogen Receptor (ER)-positive and Human Epidermal Growth Factor Receptor (HER)-2-negative breast cancer patients.

Although molecular oncology has significantly optimized prognosis for many tumor subtypes, for those tumors of which the tumorigenesis process is still largely unknown, targeted treatment options leading to long-term disease control are still lacking. Both pancreatic ductal carcinoma and glioblastoma are tumors of which the tumorigenesis process could not yet be defined, and thus, these tumors still have a dismal outcome. As a consequence, only limited progress has been made in increasing the 5-year overall survival of these tumors. Regel et al. [5] describe the precision medicine initiatives for patients’ pancreatic ductal carcinomas, allowing preclinical research and extensive molecular profiling of patient-derived tumor tissue and blood. These research initiatives may lead to a better understanding of pancreatic ductal carcinoma and the development of new biomarkers and innovative treatment strategies. In glioblastoma, multiform extensive single-cell sequencing studies have shown a large intra-tumoral heterogeneity, in which a single tumor consists of multiple tumor clones, with significant genetic and epigenetic differences. Therefore, targeted therapy strategies, inhibiting the activation of specific oncogenes, have not been successful so far. In these molecular heterogeneous glioblastomas, targeting the tumor microenvironment seems to be an interesting strategy. Immune cells are an important component of the tumor microenvironment. Like multiple other tumors, glioblastomas are able to evade an effective immune response. However, immune checkpoint inhibitors, which are known to be able to create an immune permissive immune environment by the activation of infiltrating T cells, are not effective in glioblastomas. Weenink et al. [6] summarize the available data about the unique composition of the glioblastoma-specific immune environment. This increases the understanding of why clinical trials with known immune modulators have not shown an increase in overall survival so far. In this paper, new treatment strategies are also proposed in which different immune modulators are combined. As the blood–brain barrier may limit the intratumoral availability of systemically administered compounds, Uckun and colleagues [7] describe a way to bypass the blood–brain barrier. They show that convection-enhanced (CED) intra-tumor administration of the RNA therapeutic OT101 inhibits the immunosuppressive actions of transforming growth factor beta 2 and this then results in clinically meaningful single agent activity. At the same time, several CED procedures and device-related complications are identified, and therefore, risk mitigation strategies are needed to investigate how to move forward in this field of expertise. 

Moreover, in hematologic malignancies, advances in treatment strategies have been made. These advances in treatment strategies not only focus on the development of novel targeted therapies but also on combining interesting cytotoxic chemotherapeutics in order to improve the prognosis of difficult-to-treat hematologic malignancies. Recurrent acute myeloid leukemia (AML) still has a dismal prognosis and treatment options are limited. The phase IB study results of Uckun et al. [8] show that the combination of the cytotoxic chemotherapeutics combrestatin A1 diphosphatase (OXi4503) and cytarabine (ARA-C) is generally well tolerated in older adults with relapsed AML. From this study, further clinical validation of this combined chemotherapeutic approach in the treatment of AML is warranted.

In addition to the use of molecular characteristics of tumor cells, clinical tumor features can also be valuable biomarkers, thereby guiding patient- and tumor-tailored treatment. Alonso-Alvarez et al. [9] use tumor features for tailored treatment in patients with large B-cell lymphoma. In a retrospective study, bone marrow infiltration in patients with diffuse large cell B-cell lymphoma was correlated with poor prognosis, suggesting that this patient population might need intensified treatment strategies beyond the current standard of care.

PM in oncology is not limited to optimizing anticancer therapies but can also be utilized in tailoring the supportive care of patients diagnosed with malignancies. Adequate cancer-related pain management is of utmost importance to increase the quality of life of all patients enduring anticancer treatment, in particular during end-of-life stages in which no more anti-tumor treatments are available. Opioids are mostly used for chronic pain management in cancer-related pain. Interpatient heterogeneity in opioid-related side effects and treatment effectiveness impaired optimal pain relief. Bugada et al. [10] review the pharmacogenomics data on genetic variants and opioid response and how this may be used to fine-tune opioid prescription and then optimize analgesic treatment strategies. 

In conclusion, this Special Issue on Personalized Medicine: Recent Progress in Cancer Therapy highlights recent advances in personalized and precision medicine in cancer and its accompanying symptoms. The various contributions in this Special Issue demonstrate how the fine-tuning of predictive models will not only enable the ongoing development of tumor-tailored treatment approaches but, at the same time, will also improve prognostic models. This, in the end, will result in and facilitate a shared decision towards a patient-tailored treatment plan.
